# Version control of pathway models using XML patches

**DOI:** 10.1186/1752-0509-3-34

**Published:** 2009-03-17

**Authors:** Peter Saffrey, Richard Orton

**Affiliations:** 1 Department of Computing Science, University of Glasgow, Glasgow, G12 8QQ, UK

## Abstract

**Background:**

Computational modelling has become an important tool in understanding biological systems such as signalling pathways. With an increase in size complexity of models comes a need for techniques to manage model versions and their relationship to one another. Model version control for pathway models shares some of the features of software version control but has a number of differences that warrant a specific solution.

**Results:**

We present a model version control method, along with a prototype implementation, based on XML patches. We show its application to the EGF/RAS/RAF pathway.

**Conclusion:**

Our method allows quick and convenient storage of a wide range of model variations and enables a thorough explanation of these variations. Trying to produce these results without such methods results in slow and cumbersome development that is prone to frustration and human error.

## Background

The use of computational modelling is becoming widespread within the biological community. Models are applied to a diverse array of problems and are now a standard analysis technique used both in academia and industry.

Although modelling is now widely used, the methodology that supports modelling remains underdeveloped. In particular, a model will often develop and change over time, giving rise to many model versions but there is very little published work on model version control. Some aspects of model version control are similar to the established field of software version control. However, there are a number of significant differences that mean a separate treatment is needed.

Many of the differences between software and model version control have at their core a difference in aims. The aim of a piece of software is to address a specific problem, described by its requirements. The aim of a model is the far more vague goal of understanding a biological system, sometimes expressed as specific questions (such as "does this set of reactions provoke a sustained or transient response?"), sometimes as how behaviour might change under different conditions and sometimes as a more general exploration of system properties.

This contrast between the convergent aims of a software project and the more divergent aims of a modelling project make the version control needs fundamentally different. These differences include the need to manage combinations of model versions, giving rise to a much greater branching factor. There is also a need to maintain a larger number of alternatives that are still relevant at any given time.

To address these differences, we have devised a flexible patch-based version control system that differs from existing software patching applications. Each patch represents a modification to an existing model: an addition, deletion or replacement of existing pieces. Patches can be applied in combination while retaining the original model structure. This allows later patches to work on the original system, or on a configuration created with existing patches. This flexible approach allows a modeller to rapidly explore a wide variety of model configurations without overwriting any previous ideas. We have applied the system to models expressed in SBML [1], to take advantage of the broad support for the language and the regular structure provided by XML documents. Working with XML models also means that our system could be easily extended to support other XML modelling tools, such as CellML [2].

In this paper we present details of our patch based system along with a prototype implementation. Our method is generic enough to apply to many areas of computational modelling. However, as a motivating example we have developed our methods in conjunction with a specific system: the EGF pathway. We illustrate the method with reference to the EGF pathway in a variety of versions.

### Software and Modelling Version Control

#### Software Version Control

**Figure 1:**
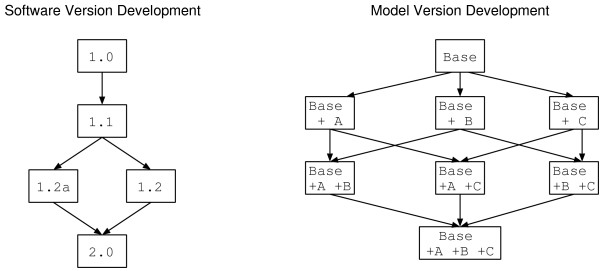
**Software and modelling version development**. Software development (on the left) tends to have a linear progression, with the aim of a single 'best' outcome. By contrast, model development (right) is a more divergent and exploratory task, encompassing various hypotheses concerning which parts of a pathway are relevant under different conditions.

Software version control is a mature and established discipline of software engineering. A characteristic progression of versions is shown in the left hand diagram of figure 1. The main features of this progression are:

• Versions proceed in a roughly linear fashion working towards a single set of requirements. Requirements may change but at any one time there is only one set.

• Each new version represents an improvement over (and usually replacement of) previous versions.

• Branching does occur, but features from different branches are usually folded back into an overall 'best' version.

Software version control tools, such as CVS [3], SVN [4] and Git [5], are designed to support these characteristics of software development with features including distributed access and the ability to fork new versions and merge these together.

#### Model Version Control

Although a pathway model is often a software artifact, there is little published material on using version control systems to track model version changes. There are a number of obvious distinctions between the progression of a pathway model and the progression of a software project. A characteristic progression of versions is shown in the right hand diagram of figure 1. The main features of this progression are:

• A model starts as a simple base and then a number of possible extensions to the pathway are suggested, each of which are largely independent of each other.

• Each extended version represents part of the pathway which is only present under certain conditions. An extension may also represent an investigation into whether a particular extra section of pathway is important or not. Each new version may represent the same part of a pathway under a different hypothesis, but is not necessarily an improvement or replacement for another hypothesis.

• Branching generates a combinatoric effect; each new extension can be applied to all the previous combination of extensions.

It may be possible to manage such a progression using a software version control system, but it would not be a natural fit. Each combination of extensions would be assigned a version number and a user would have to continually track back and forth along this timeline to locate the setup they were interested in. Applying a new extension to an existing set of combinations would require a good deal of extra work.

### XML diff and patch

A variety of systems already exist to record differences in XML files and allow these changes to be duplicated. A good review of these technologies can be found in [6]. Several algorithms [7,8] have been developed to recognise XML changes (known as a "diff") and record these changes. There are implementations of these techniques from IBM [9] and others [10].

### Simulink

Simulink is the modelling component of the mathematical toolkit Matlab [11]. Simulink allows model design based on the connection of components and provides integration with version control systems, so that each component can have its own version history.

Simulink is a flexible and powerful system, but the version control mechanisms are still based on traditional software version control; the patch-based combinatoric modification of models we propose is not supported.

## Results

### Case Study: EGF pathway

**Figure 2:**
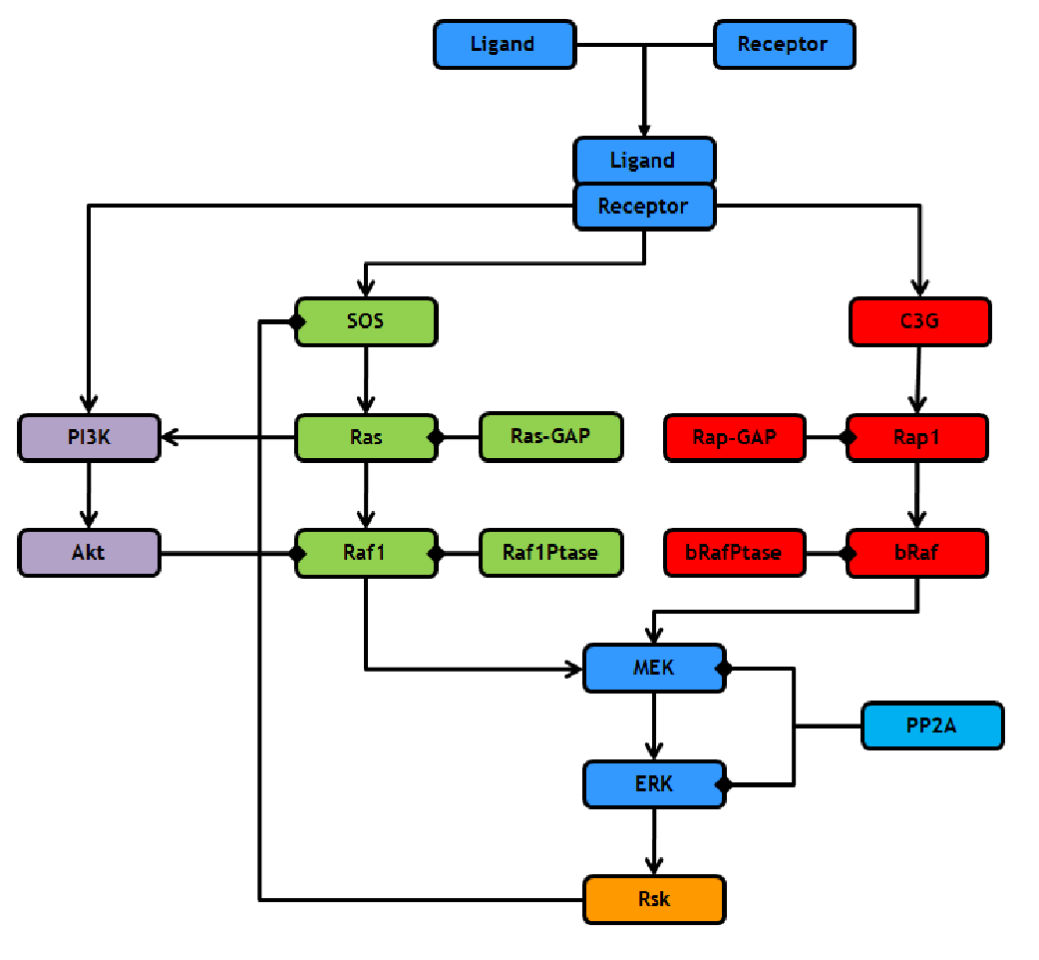
**Schematic of the receptor activated ERK pathway**. This schematic has been split into a number of distinct sections or patches which are: (1) Blue: the core patch consisting of ligand/receptor binding and the core ERK pathway; (2) Green: the Ras pathway which links bound receptors to ERK activation; (3) Red: the Rap1 pathway which links bound receptors to ERK activation; (4) Orange: the negative feedback loop from ERK to SOS via Rsk; and (5) Purple; the Akt pathway. In the schematic, links which end in arrows represent activating reactions whilst links which end in diamonds represent deactivating reactions.

To demonstrate our system we have tested it on a suite of models based on the EGFR/MAPK system. The Mitogen Activated Protein Kinase (MAPK) pathway is at the heart of a molecular signalling network that governs the growth, proliferation, differentiation and survival of many, if not all, cell types [12-14]. It is deregulated in various diseases ranging from cancer to immunological, inflammatory and degenerative syndromes, and thus represents an important drug target. Perhaps the most important and intensively studied MAPK pathway is the Extracellular-signal Regulated Kinase (ERK) pathway, which is typically initiated by the activation (via ligand binding) of cell surface receptors, such as the Epidermal Growth Factor (EGF) or Nerve Growth Factor (NGF) receptors (Figure 2). In essence, there are two distinct pathways leading from activated receptors to ERK activation, the Ras pathway which proceeds via SOS-Ras-Raf1 and the Rap1 pathway which proceeds via C3G-Rap1-bRaf. Activated ERK has numerous targets in both the cytoplasm and nucleus, including numerous transcription factors, and can therefore directly effect gene expression and influence cellular outcome. In addition, ERK is able to phosphorylate SOS (via Rsk) thus forming a negative feedback loop within the pathway [15,16].

One of the most common cell lines used to investigate ERK signalling from growth factor receptors is the PC12 (rat pheochromocytoma) cell line. In PC12 cells, EGF stimulates a rapid but transient activation of ERK, peaking at 5 mins and returning to basal levels at 30 mins, leading to cellular proliferation [17,18]. In contrast, Nerve Growth Factor stimulates a sustained activation of ERK leading to the neuronal differentiation of PC12 cells [17,18]. There is now compelling evidence that the duration of the ERK signal governs whether PC12 cells proliferate or withdraw from the cell cycle and differentiate into a neuronal phenotype [17,18]. Although the PC12 system has been well studied, it is still unclear how different ERK signal dynamics can be robustly controlled by different upstream receptors. Furthermore, there is a currently some debate as to which of the two pathways (Ras and Rap1) is utilised by the different receptors to activate ERK and relay their signal. Some groups have reported that both EGF and NGF can only use the Ras pathway [19,20], whilst others report that NGF but not EGF can also use the Rap1 pathway [21], whilst another group has reported that both NGF and EGF can use both the Ras and Rap1 pathways [18]. Over recent years the computational modelling of biological systems has become increasingly valuable and there are now a wide variety of models of the ERK pathway available which have led to some novel insights and interesting predictions as to how this system functions [22]. However, none of these studies utilised a modular or patch approach in their model design and analysis. This is unfortunate, given that the ERK pathway can be utilised by different receptors with different properties, and that there are different pathways that can be used by receptors to activate ERK. A patch system would enable one to rapidly build different versions of a receptor system composed of different pathways and feedback loops, as well as enabling the rapid application of the system to different receptors with different properties. This would enable one to rapidly investigate which version best reflects the available biological data, which pathways (or patches) are critical for relaying the signal, and also enable the easy comparison of different receptors. To demonstrate the potential of our patch approach, we took the computational model of the ERK pathway developed by Brown et al. (2004) [23] and split it into a number of distinct patches (Figure 2). We investigated the debate surrounding the use of the Ras and Rap1 pathways in NGF receptor signalling by utilising the patch approach to generate and compare three different models of the receptor system. All three models included the core ERK patch (blue) and then the following model specific patches:

1. Ras Model: This model included the Ras pathway patch (green) and the SOS feedback patch (orange).

2. Rap1 Model: This model included the Rap1 pathway patch (red).

3. Ras & Rap1 Model: This model included the Ras pathway patch (green) and the SOS feedback patch (orange), as well as the Rap1 pathway patch (red).

**Figure 3:**
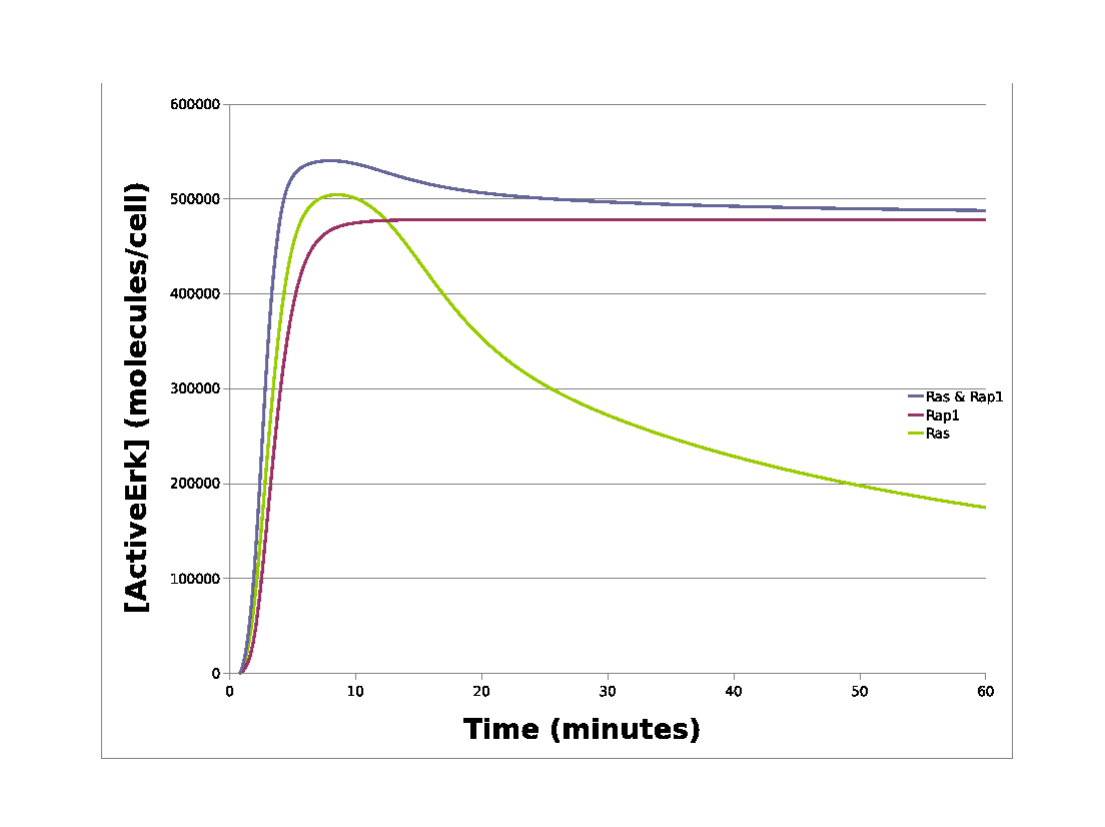
**Simulations of the NGF receptor system**. This chart contains simulation traces from various NGF receptor system models. The x-axis represents time in minutes whilst the y-axis represents the concentration of active ERK in molecules/cell. The blue line represents the simulated level of active ERK from the original Brown NGF model with both the Ras and Rap1 pathway patches, whilst the red line represents simulated active ERK with only the Rap1 pathway patch, and the green line represents simulated active ERK levels with only the Ras feedback patch. As can be seen, the Rap1 pathway patch is required to achieve a sustained ERK signal as the Ras pathway patch alone is only capable of producing a transient signal.

We first created the Ras model by using our Patch Tool to select our core ERK patch (blue) along with the Ras (green), and SOS feedback (orange) patches and subsequently launched and simulated the model in Copasi. We then used our Patch Tool to create the Rap1 model by deselecting the Ras and SOS feedback patches and selecting the Rap1 patch instead, then re-launched Copasi with the new model whilst maintaining our original simulation and plot settings. The Ras & Rap1 model was then created and simulated in a similar fashion. As can be seen in Figure 3, the Ras model produces a transient active ERK response whereas the other two models, which both include the Rap1 pathway, produce a sustained active ERK response. As NGF is well known to stimulate the sustained activation of ERK in PC12 cells, our simulation results suggest that the NGF receptor must signal via the Rap1 pathway in order to achieve this. Overall, this example illustrates the potential of our patch approach as it enables one to rapidly create different versions of a system to investigate the role that entire pathways play in the dynamics of signalling and investigate which one best reflects the available biological data. To accomplish this manually can often be a tedious affair where reactions and species are manually deleted to create new model files which are individually named and saved. However, if an error is found or an update/expansion is required, all of the saved files containing the different model versions may well need to be changed, whereas with our approach only the corresponding patch will need to be changed once. The close integration of our Patch Tool with the simulation tool Copasi is also extremely useful as once patches have been selected the model can be rapidly simulated and analysed, especially as both simulation and plot options are maintained as models are updated.

**Figure 4:**
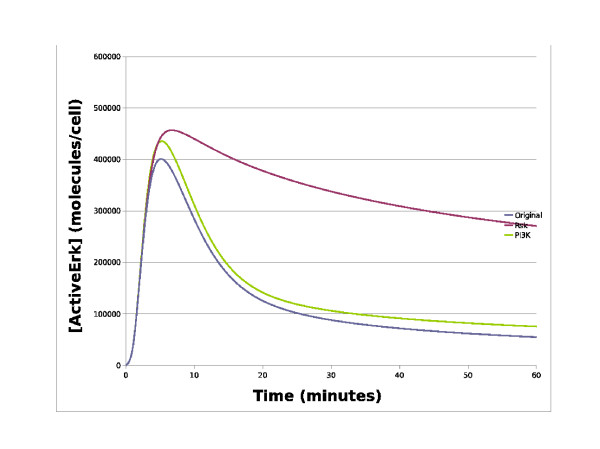
**Simulations of the EGF receptor system**. This chart contains simulation traces from various EGF receptor system models. The x-axis represents time in minutes whilst the y-axis represents the concentration of active ERK in molecules/cell. The blue line represents the simulated level of active ERK from the original Brown model, whilst the red line represents simulated active ERK levels with a Rsk knockout, and the green line represents simulated active ERK levels with a P13K knockout. As can be seen, knocking out the PI3K has little effect on active ERK whilst knocking out Rsk has a dramatic effect with the signal switching from a transient to a sustained response.

The patch approach can also be used to create dependent patches which are patches that can only be introduced into a model when a specific patch is already present. This is a useful feature when one wants to apply changes to an existing patch, such as kinetic parameter changes or species knockouts. In biological terms, such species knockouts could represent the available RNAi's to knockout proteins by knocking down gene expression, enabling one to investigate which proteins play the biggest role in transducing the signal. One interesting area of current research is focussed on how the EGF receptor system can be manipulated to give a sustained rather than a transient response [24,25]. Using our Patch Tool to investigate this, we created two species knockout patches which knockout Rsk and PI3K, respectively. As can be seen in Figure 4, knocking out PI3K has little effect on the active ERK plot whilst knocking out Rsk has a dramatic effect with the active ERK signal switching for a transient to a sustained response. Knocking out Rsk effectively knocks out the negative feedback loop from ERK to SOS, which suggests that this feedback loop is essential for signal termination. Whilst knocking out PI3K effectively knocks out the whole Akt pathway, as no other species can activate Akt, which suggests that the Akt pathway plays little role in either signal initiation or termination.

**Figure 5:**
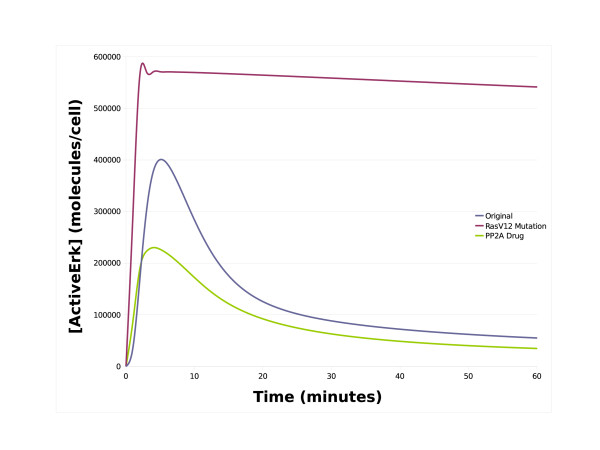
**Simulations of cancerous mutation**. This chart contains simulation traces of the EGF receptor system model with and without a RasV12 cancerous mutation. The x-axis represents time in minutes whilst the y-axis represents the concentration of active ERK in molecules/cell. The blue line represents the simulated level of active ERK from the original Brown EGF model with the normal Ras pathway patch, whilst the red line represents simulated active ERK with a cancerous RasV12 pathway patch. As can seen, swapping the normal Ras patch for a cancerous patch results in the constitutive activation of ERK which would lead to uncontrolled cell growth. The green line represents the simulated level of active ERK with the cancerous RasV12 patch and a hypothetical drug which increasing the catalytic activity of PP2A by a factor of 10. As can be seen, the drug is successful at treating the cancer as it brings active ERK down to basal levels.

Our patch approach and tool can also be used to create patches that are mutually exclusive which is a useful feature when investigating cancerous mutations. The ERK pathway has long been associated with cancer as mutations to various proteins in the pathway are well known to result in the constitutive activation of ERK leading to uncontrolled cell growth [26-28]. For example, one of the most common Ras mutations is a glycine to valine mutation at residue 12 (Ras^*V*12^) which renders Ras insensitive to inactivation by Ras-GAP and thus locked in the active state [26]. To illustrate the potential of our patch approach, we constructed a cancerous Ras pathway patch containing a constitutively active Ras and made it mutually exclusive with the standard Ras pathway patch. As can be seen in Figure 4, swapping the standard Ras pathway patch for the cancerous Ras pathway patch results in the active ERK signal switching from a normal transient response to a constitutively active response. This example illustrates the potential of the patch approach in investigating the effects of diseases such as cancer where patches representing normal and mutated/modified conditions can be created. One can imagine a situation where patches representing the different cancerous mutations have been generated along with patches representing the most common drug treatments allowing one to test which drug/combinations are the best treatments for each of the different forms of cancer. To illustrate this, we created a parameter change patch to represent a hypothetical drug capable of increasing the kcat value of PP2A by a factor of 10. This means that one can simply select the drug from the list of parameter patches and then simulate the model to analyse it effects. As can be seen in Figure 5, such a drug appears to be an effective treatment against cancers caused by Ras^*V*12^.

## Discussion

### Future Work

#### Standardised Tools

For simplicity and convenience, our tool was based on an in-house implementation of the xml-diff techniques. However, it would be good software engineering practise to base our tool on an established XML technology such as [9].

#### Version Control and Source Code Management

In this paper, we are concerned specifically with version control: how to allow a modeller to extend and change their model and revert to previous versions if necessary. This is distinct from source code management (SCM), which provides a group of programmers with a means to collaborate on a software project by managing the source code, although most SCM systems include a version control facility. Our work could easily be extended to encompass elements of source control management. The base model and patches could be uploaded into a SCM system such as SVN, which would allow users to upload new patches, amend existing patches and access the entire set of patches from a collaborative project. The difficulty with such an approach is defining the granularity of a model amendment. When a change is made to a model, should this be change to the base model, to one of the existing patches or an entirely new patch?

The view of the authors is that an XML patching system should be seen as complementary to existing SCM approaches. XML patches make hypothesis testing and combinatorial model changes far easier to manage but are not a replacement for disciplined use of an SCM in a collaborative environment.

#### Model Repositories

The issue of model granularity also arises when deciding how to submit a patch-based model to a model repository, such as Biomodels.net [29]. Many patch based models will not have a complete amalgamated version since removal patches may remove some of this amalgamated behaviour. One possibility is to upload a number of patch combinations for the model representing some more important configurations. Whether this is feasible will depend on the final number of important patch combinations.

Ideally, it should be possible to upload a base model and set of patches to allow other database users the flexibility to patch and use the model as intended. In this case, a patch standard should be devised with a reference implementation to allow model databases to support patch upload and application. This would be a more long-term development for this work.

#### Model Provenance

It might also be useful to include further methodological provisions to document model development and provenance. The plots resulting from a model run should be stored along with the configuration of patches used to generate those plots. These model reports should be stored in a database for search and retrieval. This infrastructure would leverage the patching approach to improve the efficiency of model development, as described in [30,31].

However, we believe that the best way to drive further work is to be motivated by ongoing projects. We are working with members of the SIMAP (Simulation modelling of the MAP kinase pathway) project at Glasgow to develop patching tools specific to the modelling needs of that project to develop the patching approach as it can be most useful to active work.

#### Element Name Clashes

Our patch system relies on unique element identifiers to make unambiguous references into each XML document. In larger projects with many contributors, the patch system should provide some further support to help avoid name clashes.

As a first step, the patch-set should include a name-space summary that shows what names are present in the model and in which parts of the model, base or patches, each name appears. The patch system should also allow annotations to be added to this name-space to provide a more detailed description of each name and how it differs from similar names. New patches should refer to this name list and warn where name clashes exist.

Ideally, the names used in a model should be based on a controlled ontology, similar to the Gene Ontology [32]. This would reduce the possibility of ambiguity to a minimum, make it clear what is referred to by each model element and facilitate interoperability between models.

#### Other SBML Features

Our case study only addresses modifications to species, reaction and parameter elements. However, alterations to other SBML elements should be accommodated. For example, introducing SBML events would be an addition patch of the chosen events. Altering the triggers for these events would be replacement amendments for those triggers.

In some cases, the introduction of these new elements may constitute a broader structural change to the model, which would require treatment outside of the patch system (see below).

#### Structural Changes

The XML patching system works well where new conceptual features, such as pathway sections, are introduced and removed from a system. However, it is not so appropriate when a model undergos a large structural change.

For example, if an existing model is converted to use SBML compartments, this would mean assigning all species to a compartment, so an appropriate patch would represent the entire model changing. In this case, it would be easier to start again with a fresh model of the new structure.

Once a model is built around compartments, new patch sets may be needed to represent common operations on compartment models. These could still be based on the addition, deletion and replacement amendments, but these may need to be mixed in a single patch set. For example, a species may be moved from one compartment to another – a deletion and addition amendment in the same patch set. This would necessitate dependency checking to ensure that other patches do not attempt to delete this species after it has been moved somewhere else.

#### Managing Other Model Types

CellML is similar in structure to SBML and the addition, deletion and replacement patch approach should be applicable here. New CellML components can be added using addition patches or existing ones augmented or reduced using addition and deletion patches. The component framework of CellML might allow a broader application of the patch approach. An addition could be used to represent the insertion of a component and this same patch may be used to insert this component into a variety of base models. Some additional information may be required to tailor the component interface to a variety of models, but it may be possible to implement a component approach to modelling in this way.

As with SBML, deeper changes may cause alterations across several elements; these can be represented with a combination of addition and deletion patches but may require dependency checking not to break the application of other patches. Again, as with SBML models, deep structural changes might be better represented with an entirely new model.

The addition-deletion-replacement scheme might also work effectively to manage a set of model versions in a broader environment – a Matlab model, or one written in C++ for example. These generic models can still be represented as a base and a set of changes, although the implementation of identifying and applying changes will need to address the model representation.

## Conclusion

We have presented a version management system for pathway models. We have shown how using SBML documents can allow the application of separate model amendments in combination by using an XML patch system. We have also presented an implementation of these ideas and their application in a case study.

## Methods

### XML Patching Implementation

Our XML patching implementation is based on the Fast Match Edit Script method described in [7]. We have implemented a simplified version of this algorithm to make the system more lightweight and give us fine-grained control over the way differences are detected and recorded as patches.

Although the patch technology we describe here is generic, we have developed the system specifically for use with pathway models. During this section we will describe how each concept applies to these models.

#### Patch generation

A patch represents a change or *δ *between a base system *B *and a base system with a single amendment *C*: $B\overset{\delta }{\underset{}{\to }}C$. Patch generation is a function that takes as input a base and a changed system and returns a patch *δ *that describes how to alter the base system to look like the changed system.

There are three types of patch that can be identified during patch generation: *addition*, *deletion *and *replacement*. In each case, an xpath [33] expression is used to specify whereabouts in *B *the change takes place. Each xpath references the *id *attributes used in SBML documents to make sure the referenced position is unambiguous.

Some tools like Copasi produce models using anonymous numbered id attributes such as 'id3'. To prevent name conflicts when new patches are created, we process models so that each element take its name as the id. This method is effective so long as the modeller does not use the same name to model different species and is sufficient for a prototype implementation. In larger modelling projects with many contributors, or where a model is passed from one modeller to another, the possibility of name clashes increases. This will be discussed further in the Future Work.

In a deletion patch, the xpath specifies the XML node to be removed. An addition patch specifies the XML node to be added at the given xpath. A replacement patch specifies the XML node which should be used as the replacement for the node at the given xpath.

A replacement patch could also be represented by a deletion and an addition, but dividing it into two patches makes the function of the patch less clear and less efficient. A replacement patch of the complete document would constitute a *δ *between *B *and *C *but would again be less clear and less efficient; the xmldiff algorithm builds the smallest possible replacement patch, with the minimum patch size being one complete XML element.

XPath supports referring to specific attributes, so it would be possible to use patches at this level of granularity. However, element level addition and replacement patches can contain complete well-formed XML to insert at this point. To implement attribute additions and replacements requires making a special case, since an attribute on its own needs extra structure to become well formed XML. Any attribute are still possible with element level patches and can be implemented without augmenting the schema to include special cases.

These patch operations are similar to the Delta Update Language described in [6] but without the *move *operation.

#### Patch files

Individual addition, deletion and replacement amendments can be grouped together into patch files. Each patch file represents a number of amendments to transform a base system into the base system with some collection of changes. In theory, patch files could contain a mixture of addition, deletion and replacment changes. However, this raises the possibility that individual amendments contradict each other: for example, a deletion may delete the point at which an addition should occur. For simplicity, we only allow each patch file to contain amendments of one type. This gives rise to patch files that represent either a set of additions, or a set of deletions, or a set of replacements.

In a pathway model, an addition patch file represents a new part of the pathway, including new substrates and their associated reactions. A deletion patch file represents a knockout, where one or more substrates have been removed from the system.

A replacement patch file represents altering the kinetics of one or more reactions, providing a new set of parameters. The change patch file contains one change amendment for each parameter. Each amendment refers to the XPath for that particular parameter and contains a complete XML element representing that parameter, with the new value as the "value" attribute. Collecting a set of parameters into a single file and allowing a number of such sets is similar to the concept of Parameter Run File presented in [30].

#### Patch dependencies

In some cases, it is desirable to impose dependencies on a set of patches so that one patch can be applied only after another. It is also possible for one patch to exclude the use of another.

In a pathway model, a particular knockout will only make sense in the presence of the targetted substrates and reactions. A knockout patch can therefore depend on a new pathway patch that introduces these substrates and reactions to the base system.

A new pathway patch may exclude another if these represent different conditions of the same pathway. For example, one patch may represent the normal condition of this pathway and another the cancerous condition; only one version of the pathway should be applied for a particular configuration.

#### Patch Schema

An outline of the patch schema is as follows:

• **Changes **Top level element. Attributes: name of patch.

**- Description **Textual itemize of patch.

**- Dependency List **A list of dependencies.

* **Dependency **Attributes: name of patch upon which this patch depends

**- Exclusion List **A list of exclusions.

* **Dependency **Attributes: name of patch which cannot be applied of this patch is applied.

**- Change List **List of patches.

* **Change **Attributes: type (addition, deletion or replacement); xpath to change location. If this is an addition or replacement patch, the elements beneath this node will be what should be inserted at the xpath provided.

#### Summary: patches applied to pathway models

Table 1 provides examples of how the patch system might apply in a pathway context.

**Table 1:** Patch applications in pathway modelling

**Patch feature**	**Example application**
Addition patch	Adding a substrate or reaction

Deletion patch	Removing a substrate or reaction

Replacement patch	Changing a reaction rate

Addition patch file	Adding a new pathway of reactions and substrates

Remove patch files	A knockout of a substrate and its associated reactions

Replacement patch files	Changing a set of reaction rates

Dependent patches	A knockout applied to a particular pathway

### Tool overview

**Figure 6:**
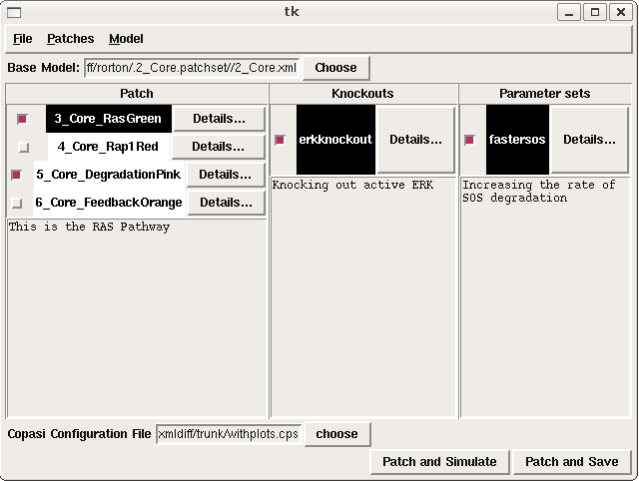
**Model version control tool screenshot**. This figure is a typical view of our version control tool in use. The top text box shows the base model to which the various patches can be applied. The left hand panel shows the pathways (addition patches) available. The central panel shows knockout (deletion patches). The right hand panel shows parameter sets (change patches). A text box at the bottom shows a Copasi file storing configuration settings for models (such as variables for plotting), which will be applied to Copasi on choosing the 'patch and simulate' button.

To demonstrate the efficacy of our approach, we have implemented a prototype and evaluated it on a case study (see Case Study section). The tool was implemented in Python using the pxdom XML library and Tkinter to provide a graphical user interface. Figure 6 shows a screenshot of the tool in use.

#### Copasi Integration

Our tool operates on biological models expressed in SBML. To obtain these models and to simulate models after applying the patches, we have integrated our tool with the popular SBML simulation tool Copasi [34]. Patches are generated from Copasi-generated SBML files and Copasi can be automatically launched on a model with the selected patches. We also allow Copasi configuration information, such as variable plots, saved in a Copasi file to be used for successive patched systems.

#### Use case

A basic use-case for the tool is as follows:

### Setup pathway patches

1. Using Copasi, build a 'base' model and export as SBML, containing the fundamental elements of the overall system. Import this base system into the patch tool

2. Also using Copasi, build an extended model representing the base system along with an extra pathway. Export this as SBML and import into the patch tool. Add a name and description for this pathway.

3. Repeat step 2 for additional pathways. In each case, build an SBML file that represents the base file with the substrates and reactions of a new pathway added.

### Setup knockouts and parameter sets

1. If desired, add one or more knockouts. Each knockout can apply to one or more pathways or to the base system. The tool provides a dialog box to select the substrates to be knocked out.

2. If desired, add one or more parameter sets. Parameters sets can apply to one or more pathways or the base system.

### Choose patches to apply

1. Select the pathways, knockouts and parameter sets for the desired model configuration. If a knockout or parameter set depends on a pathway, this pathway also must be selected.

2. Save the chosen model configuration to SBML or automatically launch it in Copasi.

## Availability and requirements

The software prototype described in this paper is available.

**Project Name **XML patching tool.

**Project Home **Page The tool is available at  or see additional files 1. A README file is included with the distribution. There is also a sample set of patches for testing to be found in  or see additional files 2.

**Operating System **The tool has been tested on Ubuntu Linux 8.04 and Mac OS 10.4, but should work on other platforms that support Python.

**Programming Language **Python.

**Other requirements **Python-xml libraries; pxdom; an SBML compliant modelling tool such as Copasi.

**License **GNU GPL.

## Authors' contributions

PS had the original idea and built the tool. RO provided the biological expertise and tested the software for the case study. All authors read and approved the final manuscript.

## Supplementary Material

Additional file 1 **XML diff and patch tool.** Source code for the tool described in this paper. Instructions for installation and use are included in the file. Click here for file

Additional file 2 **Sample additional file title.** A sample patch set, describing the model used in this paper. Click here for file

## References

[B1] Hucka M, Finney A, Sauro H, Bolouri H, Doyle J, Kitano H, Arkin A, Bornstein B, Bray D, Cornish-Bowden A (2003). The systems biology markup language (SBML): a medium for representation and exchange of biochemical network models. Bioinformatics.

[B2] Hedley W (2001). A short introduction to CellML. Philosophical Transactions of the Royal Society A: Mathematical, Physical and Engineering Sciences.

[B3] Concurrent Versions System. http://www.nongnu.org/cvs/.

[B4] Subversion open source version control system. http://subversion.tigris.org/.

[B5] Git – Fast Version Control System. http://git.or.cz/.

[B6] Mouat A (2002). XML diff and patch utilities. CS4 Dissertation, Heriot-Watt University, Edinburgh, Scotland, Senior Project.

[B7] Chawathe S, Rajaraman A, Garcia-Molina H, Widom J (1996). Change detection in hierarchically structured information. ACM SIGMOD Record.

[B8] Salzburg A (2005). Structure-Preserving Difference Search for XML Documents. Structure.

[B9] IBM Alphaworks XML TreeDiff. http://www.alphaworks.ibm.com/tech/xmltreediff.

[B10] Dommitt Inc. Merge Utility for XML. http://www.dommitt.com/.

[B11] Guide M (1998).

[B12] Cobb M (1999). MAP kinase pathways. Progress in Biophysics and Molecular Biology.

[B13] Widmann C, Gibson S, Jarpe M, Johnson G (1999). Mitogen-Activated Protein Kinase: Conservation of a Three-Kinase Module From Yeast to Human. Physiol Rev.

[B14] Chang L, Karin M (2001). Mammalian MAP kinase signalling cascades. Nature.

[B15] Langlois W, Sasaoka T, Saltiel A, Olefsky J (1995). Negative Feedback Regulation and Desensitization of Insulin-and Epidermal Growth Factor-stimulated p21 Activation. Journal of Biological Chemistry.

[B16] Waters S, Holt K, Ross S, Syu L, Guan K, Saltiel A, Koretzky G, Pessin J (1995). Desensitization of RAS Activation by a Feedback Disassociation of the SOS-Grb2 Complex. J Biol Chem.

[B17] Traverse S, Gomez N, Paterson H, Marshall C, Cohen P (1992). Sustained activation of the mitogen-activated protein (MAP) kinase cascade may be required for differentiation of PC12 cells. Comparison of the effects of nerve growth factor and epidermal growth factor. Biochem J.

[B18] Kao S, Jaiswal R, Kolch W, Landreth G (2001). Identification of the Mechanisms Regulating the Differential Activation of the MAPK Cascade by Epidermal Growth Factor and Nerve Growth Factor in PC12 Cells. Journal of Biological Chemistry.

[B19] Lu L, Anneren C, Reedquist K, Bos J, Welsh M (2000). NGF-dependent neurite outgrowth in PC12 cells overexpressing the Src homology 2-domain protein Shb requires activation of the Rap1 pathway. Experimental Cell Research.

[B20] Zwartkruis F, Wolthuis R, Nabben N, Franke B, Bos J (1998). Extracellular signal-regulated activation of Rap1 fails to interfere in RAS effector signalling. EMBO Journal.

[B21] York R, Yao H, Dillon T, Ellig C, Eckert S, McCleskey E, Stork P (1998). Rap1 mediates sustained MAP kinase activation induced by nerve growth factor. Nature.

[B22] Orton R, Sturm O, Vyshemirsky V, Calder M, Gilbert D, Kolch W (2005). Computational modelling of the receptor-tyrosine-kinase-activated MAPK pathway. Biochem J.

[B23] Brown K, Hill C, Calero G, Myers C, Lee K, Sethna J, Cerione R (2004). The statistical mechanics of complex signaling networks: nerve growth factor signaling. Physical Biology.

[B24] Traverse S, Seedorf K, Paterson H, Marshall C, Cohen P, Ullrich A (1994). Research Paper EGF triggers neuronal differentiation of PC12 cells that overexpress the EGF receptor. Current Biology.

[B25] Brightman F, Fell D (2000). Differential feedback regulation of the MAPK cascade underlies the quantitative differences in EGF and NGF signalling in PC12 cells. FEBS Letters.

[B26] Bos J (1989). RAS oncogenes in human cancer: a review. Cancer Res.

[B27] Davies H, Bignell G, Cox C, Stephens P, Edkins S, Clegg S, Teague J, Woffendin H, Garnett M, Bottomley W (2002). Mutations of the BRAF gene in human cancer. Nature.

[B28] Voldborg B, Damstrup L, Spang-Thomsen M, Poulsen HS (1997). Epidermal growth factor receptor (EGFR) and EGFR mutations, function and possible role in clinical trials. Annals of Oncology.

[B29] Le Novère N, Bornstein B, Broicher A, Courtot M, Donizelli M, Dharuri H, Li L, Sauro H, Schilstra M, Shapiro B (2006). BioModels Database: a free, centralized database of curated, published, quantitative kinetic models of biochemical and cellular systems. Nucleic Acids Res.

[B30] Saffrey P, Margoninski O, Hetherington J, Varela M, Yamaji S, Finkelstein A, Bogle D, Warner A (2007). End-to-End Information Management for Systems Biology. Lecture Notes in Computer Science.

[B31] Hetherington J, Bogle I, Saffrey P, Margoninski O, Li L, Rey MV, Yamaji S, Baigent S, Ashmore J, Page K, Seymour R, Finkelstein A, Warner A (2007). Addressing the challenges of multiscale model management in systems biology. Computers and Chemical Engineering.

[B32] Ashburner M, Ball C, Blake J, Botstein D, Butler H, Cherry J, Davis A, Dolinski K, Dwight S, Eppig J (2000). Gene ontology: tool for the unification of biology. The Gene Ontology Consortium. Nat Genet.

[B33] Clark J, DeRose S (1999). XML Path Language (XPath) Version 1.0. W3C Recommendation.

[B34] Hoops S, Sahle S, Gauges R, Lee C, Pahle J, Simus N, Singhal M, Xu L, Mendes P, Kummer U (2006). COPASI-a COmplex PAthway SImulator. Bioinformatics.

